# Tetanus from Anti-Variolar Vaccination

**Published:** 1915-10

**Authors:** 


**Tetanus From Anti-Variolar Vaccination.** John F. Anderson of the Public Health Service has investigated 41 cases. In no instance were either tetanus bacilli or spores found in the stock virus represented by the vaccinations. Among 585,000 army and navy vaccinations, 8 cases of tetanus developed but no relation to the vaccination could be demonstrated. Monkeys and Guinea pigs, which are susceptible to both vaccinia and tetanus were vaccinated with virus purposely contaminated with tetanus bacilli but tetanus failed to develop. Hence, he is sceptic as to the occurrence of tetanus from vaccination.

